# Cancer associated fibroblasts in tumors: focusing on solid tumors and hematological malignancies

**DOI:** 10.3389/fonc.2025.1596947

**Published:** 2025-06-23

**Authors:** Chengyun Pan, Lin Zheng, Jishi Wang

**Affiliations:** ^1^ Department of Hematology, Affiliated Hospital of Guizhou Medical University, Guiyang, Guizhou, China; ^2^ Department of Hematology, Hematological Institute of Guizhou Province, Guiyang, Guizhou, China; ^3^ Key Laboratory of Hematological Disease Diagnostic and Treatment Centre, Guizhou Province Hematopoietic Stem Cell Transplantation Centre, Guiyang, Guizhou, China

**Keywords:** cancer associated fibroblasts, microenvironment, tumor progression, hematological malignancies, heterogeneity

## Abstract

The co evolution of tumor cells and microenvironmental matrix components almost determines the series of processes involved in cancer occurrence and progression. However, many anti-cancer treatments are designed around tumor cells, neglecting the supportive role of stromal cells. Cancer-associated fibroblasts (CAFs), as the main stromal cells in tumor microenvironment, are currently considered as a key component promoting tumorigenesis, development, and regulating the transfer of tumor cells to distant locations through secretion of different growth factors, cytokines, chemokines, and the degradation of extracellular matrix. Therefore, the strategy of targeting both cancer cells and CAFs shows great potential in cancer treatment. In hematological malignancies, the role of CAFs in the progression of tumors has gradually been recently tapped. This review describes the role and functional characteristics of CAFs in tumors, mainly concentrates on the potential role of CAFs in the disease progression of hematological malignancies according to recent findings, and emphasizes the importance of CAFs as a key target to overcome tumor progression and improve treatment efficacy.

## 1 Introduction

The tumor microenvironment (TME) is a dynamic network which is composed of stromal cells (fibroblasts, endothelial cells, immune cells, adipocytes, pericytes and bone marrow-derived cells, etc.), extracellular matrix (ECM), soluble cytokines and growth factors. The occurrence and development of stromal component provide a supportive environment for a variety of cancers cells (1–3). During the occurrence and development of tumors, tumor cells and their surrounding stromal microenvironment are in close proximity, and the extensive and multi-layered “cross-talk” between them adapts TME to support tumor survival, growth and metastasis (4, 5). Targeting the TME is currently one of the directions to improve the efficacy of tumor therapy (6–9). In the treatment of hematological malignancies, part of the reason for the poor efficacy may be that the tumor cells are protected by the “educated” microenvironment, which offers a natural refuge for tumor cells to escape the killing of chemotherapy drugs and thus becomes a possible root cause of tumor progression (10–12).

Cancer-associated fibroblasts (CAFs), a major component of TME, reside in symbiotic relationship with cancer cells, supporting them to survive from cancer drugs (13–17). In the TME, CAFs are in a continuously activated state, which not only promotes the growth of tumor cells, but also secretes various cytokines, chemokines and inflammatory mediators in a paracrine manner through cell and cell interactions, initiates the proteolysis and structural modification of the ECM, provides convenient conditions for tumor cells to escape chemotherapeutics, initiates metabolic reprogramming, and finally results in tumor progression and tumor cell migration to distant locations (18–26). It is of note that CAFs have been shown to have different origins, phenotypes, and functions, while most of them contribute to tumor progression. Considering its critical role in promoting tumor progression, CAFs have recently become a therapeutic target for a variety of tumors (25, 27). In the study of hematological malignancies, recent studies have indicated that CAFs are also a vital component in promoting tumor progression (28, 29). In addition, they may become a potential target for the treatment of hematological malignancies. This review introduces the origin, activation mechanism and role of CAFs in tumors, mainly elaborates on the possible roles played by CAFs in hematological malignancies, and potential therapeutic strategies targeting CAFs, as well as elucidates the heterogeneity of CAFs based on current research status.

## 2 Origins of CAFs

CAFs, as important stromal cells in the TME, usually originate from stromal cells in the microenvironment. These interstitial cells mainly include resident tissue fibroblasts, adipocytes, pericytes and bone marrow-derived mesenchymal stem cells (BM-MSCs). In addition, both epithelial cells and endothelial cells can be activated into CAFs through interstitial transformation. Among them, normal fibroblasts (NFs) are an important source of CAFs (30, 31). NFs typically moderately express α-smooth muscle actin (α-SMA), fibroblast activation protein (FAP), fibroblast specific protein-1 (FSP-1), and vimentin, while CAFs often highly express these proteins (32). Fibroblasts are in a quiescent state under normal physiological conditions and can be activated in tissue repair and TME. During tissue repair, the transient moderate activation of myofibroblasts is beneficial for the repair of tissue integrity. However, activated CAFs in the TME can maintain a sustained state of activation and become important factors affecting tumor occurrence and progression.

BM-MSCs are another important source of CAFs (33–35). Quante et al. (33) considered that at least 25% of activated CAFs were derived from BM-MSCs. The morphological characteristics of CAFs are similar to BM-MSCs, being adherent and growing in a spindle shape. Research has shown that the two have a high degree of consistency in immune phenotype and function (35, 36). On the one hand, CAFs expresses cell surface markers of MSCs and has a certain degree of multipotent differentiation potential. On the other hand, MSCs also express certain surface marker proteins of CAFs, such as α-SMA, nestin, and vimentin, but the expression levels of CAFs surface markers, cytokines, growth factors, and chemokines are higher.

In addition, adipocytes are also one of the sources of CAFs (37, 38). The study by Simiczyjew et al. (39) demonstrated that adipocytes co-cultured with melanoma cells could exhibit fibroblast characteristics and secrete higher levels of IL-6 and serpine1, while producing less C-C motif chemokine ligand 2 (CCL2), chemokine (C-X-C motif) ligand 1 (CXCL1), and angiogenic molecules. Lactic acidosis is a characteristic of the TME, research has shown that human subcutaneous adipose-derived stem cells committed to adipocytes can acquire myofibroblast, pro-fibrotic, and pro-inflammatory phenotypes when cultured in an acidic environment (40). In the study of gastric cancer (GC), adipocytes, when co-cultured with GC cells, significantly increased the expression levels of CAFs markers FSP-1, inflammatory cytokines, PAI-1, and IL-6, while the invasiveness of GC cells was enhanced, suggesting that adipocytes can acquire the CAFs phenotype under the stimulation of GC cells, which further promotes the invasion process of GC cells (41). Moreover, epithelial cells, endothelial cells, stellate cells, and pericytes in the TME are also possible sources of CAFs (42–44), and transformed CAFs may play an important role in the occurrence and development of various tumors.

## 3 The markers and characteristics of CAFs

For the markers of CAFs, a number of intracellular, extracellular and cell surface proteins with increased expression have been used to isolate or identify CAFs, including α-SMA, FAP, FSP-1, vimentin, periostein, platelet derived growth factor receptor-α/β, and neuron glial antigen 2 (NG2), etc. In addition, there are some negative indicators which can be used to help exclude CAFs. For instance, the antibody against CD31 is employed to demonstrate a lack of endothelial cell contamination, while cytokeratin is used to exclude epithelial components. Among these markers, α-SMA and FAP have been known as the specific markers for myofibroblasts, and their high expression often indicates a poor tumor prognosis (33, 45–52). However, till the present, no unique marker can be identified to investigate the existence of CAFs. Dzobo et al. (53) conducted a database analysis and found that CAFs markers exhibit differential expression in different tumors, and the expression patterns of CAFs markers in different tumor types are also variable. CAFs are mostly characterized based on a combination of the above markers and different tumor types.

**Figure 1:**
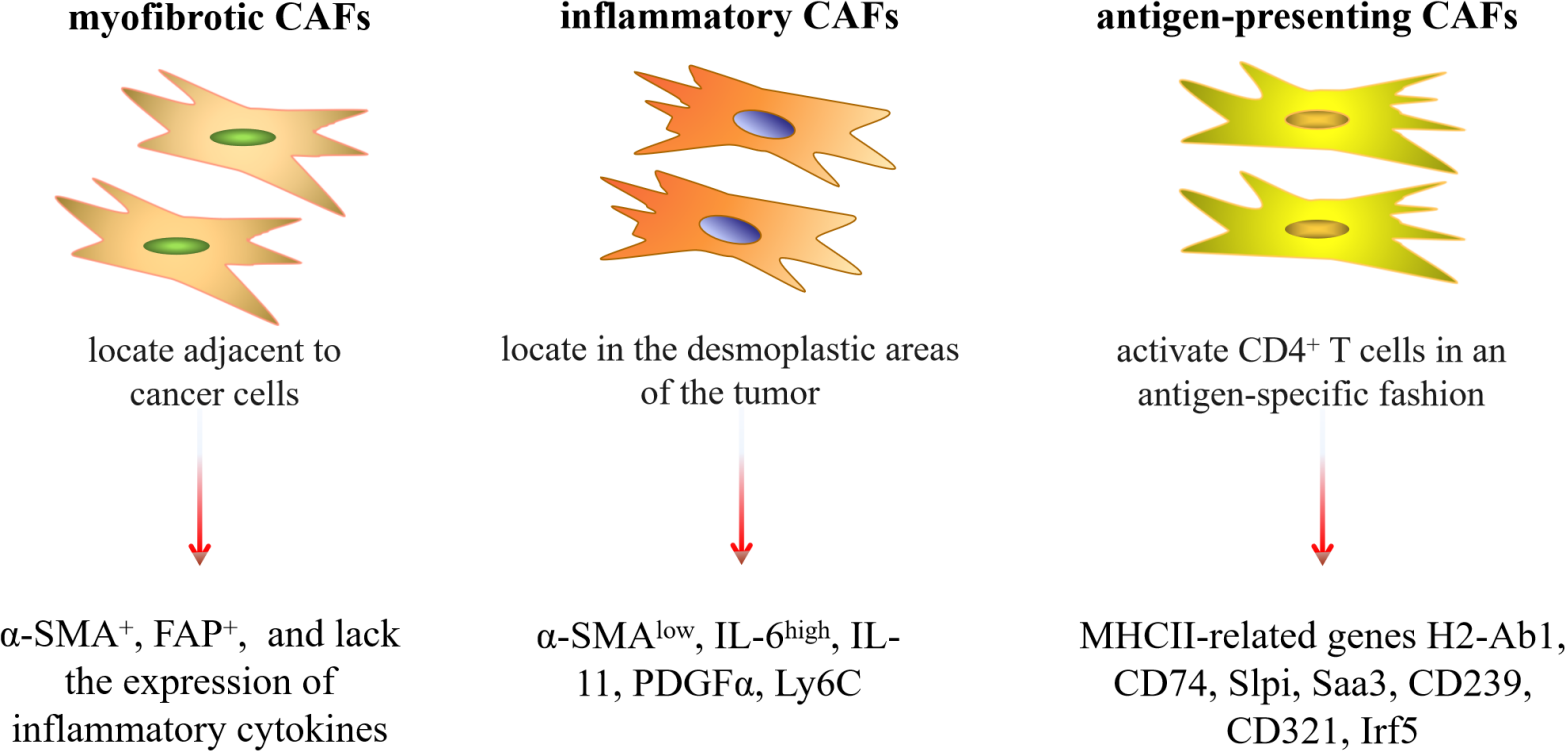
Three common subtypes of CAFs and their characteristics. Slpi, secretory Leukocyte Peptidase Inhibitor; Saa3, serum Amyloid A3; Irf5, interferon regulatory factor 5.

It is worth noting that due to the different sources of CAFs, they have various phenotypes, including the myofibrotic CAFs subtype (myCAFs) with α-SMA^+^FAP^+^ and lacking the expression of inflammatory cytokines, the inflammatory CAFs subtype with low expression of α-SMA and secretion of interleukin-6 (IL-6) and other inflammatory mediators, and the antigen-presenting CAFs subtype (apCAFs) with high expression of major histocompatibility complex (MHC) class II (MHCII)-related genes H2-Ab1, CD74 and other regulators of immune activity (27, 54–56) (**Figure 1**). Recently, reports by Cords et al. (57), Xiao et al. (58), and Zhang et al. (59) have provided a more detailed classification of the different CAF subtypes found in various tumors. Additionally, Lavie et al. (44) summarized the main CAF subtypes and characteristics of various organs in the body based on single-cell sequencing data. Because of these different subtypes, CAFs exert a variety of biological roles in tumorigenesis, progression, immune regulation, tumor metabolism and metastasis. Sahai et al. (60) suggested that a new naming framework could be constructed based on the functions of CAFs, and standardized methods could be established to detect and identify CAFs. This might lead to a more detailed and accurate classification and understanding of the characteristics of CAFs.

## 4 The activation mechanism of CAFs in tumor microenvironment

Since the activation of CAFs in the TME is a vital factor leading to malignant progression of tumors, understanding the activation mechanism of CAFs in the TME may be a key measure to find effective intervention methods to block the activation of CAFs in the microenvironment and enhance the efficacy of tumor treatment.

In the TME, to obtain invasive and tumor-promoting phenotypes, NFs and other stromal cells undergo continuous activation via different mechanisms. Cancer cells secrete cytokines and soluble components into the surrounding microenvironment, and then stimulate the recruitment and activation of fibroblasts. During this process, transforming growth factor β (TGF-β) is currently recognized as the key factor to stimulate the activation of CAFs (33, 47, 61–65), which is a cytokine essential for inducing the fibrotic response and activating the cancer stroma and can be expressed by both tumor cells and CAFs. Under the action of TGF-β, NFs or potential stromal cells undergo morphological changes and transform into CAFs, while the latter exert an important role in the occurrence, development and progression of tumors.

In addition to the classic TGF-β pathway stimulating CAFs activation, there are also some other potential factors that can stimulate CAFs activation. The study conducted by Weber et al. (66) showed that tumor cell-derived osteopontin (OPN) mediated the transformation of MSCs to CAFs and therefore increased tumor cell growth and metastasis, while the process was still dependent on myeloid zinc finger 1 and TGF-β1. In the study of GC, it was found that helicobacter pylori (Hp) infection might induce the transformation of MSCs into CAFs, contributing to the occurrence and development of GC (67). The reduced extracellular pH value may also be an important factor for promoting the conversion of MSCs into CAFs (68). Additionally, progranulin (PGRN) can directly or indirectly activate CAFs through the epithelial-mesenchymal transition (EMT) program to promote the invasiveness of ovarian cancer cells (69). A recent study indicated that overexpression of galectin-1 could induce the transformation of NFs into CAFs (70). These results suggest that the activation mechanisms of CAFs may vary in different TME, and we need to conduct categorical research on different tumors to better comprehend the activation factors of CAFs in tumors.

## 5 CAFs in solid tumors - the tumor-promoting function

CAFs have been widely studied in promoting tumor angiogenesis, facilitating tumor invasion and metastasis, enhancing tumor resistance and tumor immune escape in a variety of solid tumors. For tumor angiogenesis, tumor growth depends on the formation of new blood vessels, and CAFs can promote tumor angiogenesis through secretion or high expression of various pro-angiogenic and other factors, including vascular endothelial growth factor (VEGF) (71–73), interleukin-6 (IL-6) (72), fibroblast growth factor (FGF) (71), CCL2 (74), galectin-1 (75), milk fat globule-EGF factor 8 (MFGE8) (76), Wingless-type MMTV integration site family member 2 (WNT2) (77) and FOS-like 2 (FOSL2) (78). Meanwhile, the exosome (79), exosome microRNA (80) and extracellular vesicles (EVs) (81) derived from CAFs are also key components that promote tumor angiogenesis. These angiogenic factors derived from CAFs provide convenient conditions for the growth and metastasis of tumors. In terms of tumor invasion and metastasis, CAFs stimulate the invasion and metastasis of cancer cells by promoting epithelial mesenchymal transformation (82, 83), overexpressing its own markers (24, 48), or secreting of growth factors (23, 84), cytokines such as IL1β/IL-1R (85–87), IL-6 (88), IL-11 (89), IL-17a (90), IL-22 (91) and leukemia inhibitory factor (92), chemokine receptors (93), adhesion factors (94, 95), exosomes (96–102), and various metalloproteinases (MMPs) (23, 71, 88). In addition, CAFs secrete a large amount of ECM proteins to promote ECM synthesis and reshape tumor matrix, which is also one of the vital reasons for the formation of tumor aggressive microenvironment. These proteins mainly include collagen type I, collagen type III, fibronectin (FN) and vimentin. Among them, FN is the main ECM protein, which promotes tumor targeted migration and invasion through interacting with its integrin receptors (103, 104). Recent studies have also indicated that CAFs that overexpression of FN1 and periosteal protein can significantly promote the wound healing and invasion ability of tumor cells (105). For tumor resistance, CAFs can secrete various factors making cancer cells develop drug resistance. Among them, IL-6 secreted by CAFs can reduce the response of cancer cells to chemotherapeutics (106, 107), and stromal cell-derived factor-1 (SDF-1) secreted by CAFs (108) can stimulate the malignant progression of pancreatic cancer and mediate the development of gemcitabine resistance. CAFs can also secrete CCL5, which promotes up-regulation of androgen receptor expression in prostate cancer cells, resulting in resistance to enzalutamide treatment, and improves the expression of tumor programmed death ligand 1 (PD-L1), causing immune escape (109). In addition, CAFs inhibit ferroptosis and reduce cisplatin sensitivity in nasopharyngeal carcinoma by secreting FGF5 and activating downstream FGFR2/Nrf2 signaling pathways (110). In the study of esophageal squamous cell carcinoma, CAFs promote tumor cell growth by secreting plasminogen activator inhibitor-1 (PAI-1) and attenuate the therapeutic sensitivity of cisplatin (111). Meanwhile, the secretion of exosomes by CAFs is also one of the important factors which can mediate tumor drug resistance. The exosomes secreted by CAFs promote tumor cell metastasis and drug resistance to chemotherapy drugs by maintaining tumor cell stemness and promoting epithelial mesenchymal transformation (101). Moreover, CAFs may enhance drug resistance through inhibiting drug accumulation and combating drug-induced oxidative stress (112). For tumor immune escape, CAFs can induce immunosuppressive cell infiltration and create immunosuppressive TME, contributing to poor prognosis (105, 113–115). A high proportion of CAFs is more likely to cause distant metastasis of the tumor and present a higher level of immune invasion (116, 117). Additionally, The interaction between CAFs and immune cells in TME is also an important factor in promoting tumor progression (118). CAFs can enhance the infiltration and function of myeloid suppressor cells, T cells and other immune cells, reduce the number and activity of tumor-infiltrated cytotoxic T cells in tumor tissues, and make tumors insensitive to PD-1 treatment. Depletion of CAFs can improve the efficacy of tumor immunotherapy (119).

**Figure 2:**
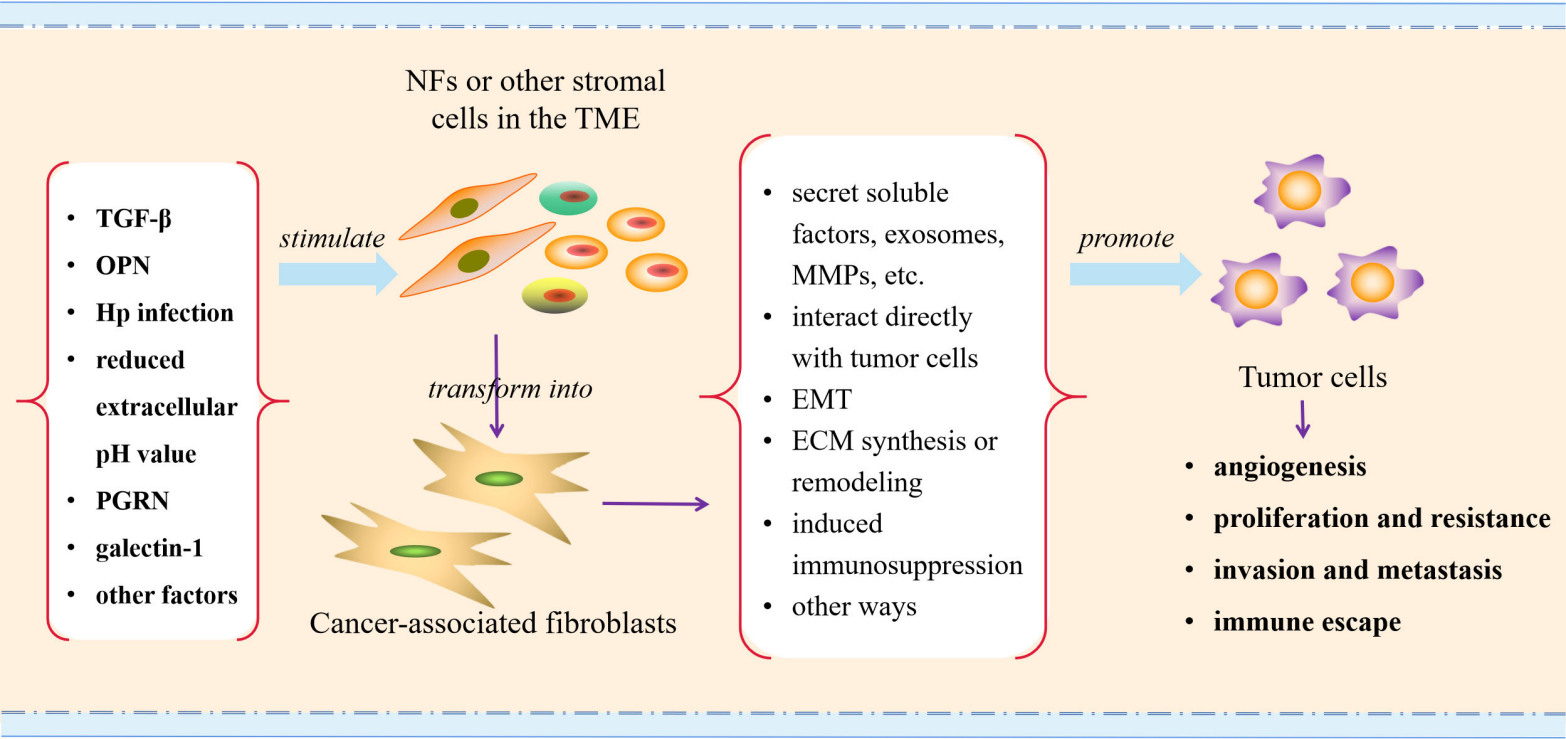
Schematic illustration of CAFs activating in TME and promoting tumor progression. TGF-β, transforming growth factor β; OPN, osteopontin; Hp, helicobacter pylori; PGRN, progranulin; MMPs, metalloproteinases; EMT, epithelial-mesenchymal transition; ECM, extracellular matrix.

In addition to the above-mentioned aspects, CAFs have been shown to alter the architecture and physical properties of the ECM, influencing tumor cells growth, migration and invasion (120–122). Meanwhile, the age-related secretion phenotype (SASP) of CAFs is also an important factor influencing tumor progression (123). Fan et al. (124) identified a new myCAFs subpopulation of aging like tetraspanning protein-8 (TSPAN8) (+) in breast cancer. TSPAN8 (+) myCAFs can resist chemotherapy by secreting SASP related factors IL-6 and IL-8, thus enhancing the stemness of surrounding breast cancer cells. Therefore, the combination of traditional tumor therapy and anti-aging drugs may also be one of the strategies to improve the effectiveness of tumor treatment. These findings demonstrate that CAFs exert an important role in the progression of solid tumors (**Figure 2**), and targeting CAFs may be an effective strategy to improve the therapeutic efficacy of multiple solid tumors.

## 6 CAFs in solid tumors - the tumor-restraining function

Although most studies have confirmed that CAFs are closely related to tumor progression, some research has also shown that CAFs have a tumor-suppressing effect. The cell surface and secretory protein meflin is expressed in cultured MSCs, fibroblasts, and pericytes. meflin-positive CAFs are related to a better prognosis in pancreatic ductal cell carcinoma. Overexpression of meflin inhibits tumor growth, while lack of meflin results in significant tumor progression and poor histological differentiation (125). In the research on lung cancer, CD200^+^ CAFs can increase the sensitivity of epidermal growth factor receptor (EGFR) gene mutation-positive lung cancer cells to gefitinib (126). Similarly, the IL-8 produced by CAFs can inhibit the proliferation of biliary tract cancers cell line OCUCh-LM1 (127). Yes-associated protein 1 (YAP1) is a protein with multiple functional domains and belongs to the Yes-related protein family. Song et al. (128) recently discovered that YAP1 can regulate the phenotype of CAFs, which can transform CAFs from the tumor-promoting subtype that promotes ECM deposition to the tumor-suppressing subtype that stimulates anti-tumor immunity, thereby increasing the treatment sensitivity of immune-checkpoint blockade. myCAF is one of the important subtypes that promote tumor progression. Bhattacharjee et al. (129) found that the collagen type I expressed by myCAF can inhibit tumor growth by collagen physically restricting tumor spread, and the absence of collagen type I can promote the growth of metastatic tumors. In the research on rectal cancer, Qin et al. (130) showed that neoadjuvant chemotherapy can significantly reshape the CAF subtypes, and the reshaped CAF subtypes regulate the TME through spatial recruitment and crosstalk, activate immunity through multiple cytokines, and inhibit tumor progression. In addition, CD143^+^ CAFs can predict better survival outcomes for colorectal cancer patients (131). In the research on malignant melanoma, it was found that CD9^+^ exosomes derived from CAFs have a significant inhibitory effect on the proliferation of malignant melanoma cells, and compared with CD9^-^ patients, CD9^+^ patients have better disease-free survival rates (132). These research results suggest that in addition to the well-known tumor-promoting functions of CAFs, they also possess potential anti-tumor functions and subtypes. These functions and subtypes should be given due attention.

## 7 CAFs in hematological malignancies

Hematological malignancies mainly include various kinds of leukemia, multiple myeloma (MM) and malignant lymphoma. With the continuous improvement of treatment programs, the five-year survival of hematologic malignancies has increased in recent years. However, whether this has translated into greater long-term survival is unknown (133). TME is an important factor affecting blood tumor survival, drug resistance and disease progression. CAFs in TME have been shown to exert a key role in promoting tumor progression in studies of acute myeloid leukemia (AML), acute lymphoblastic leukemia (ALL), chronic lymphoblastic leukemia (CLL), MM, lymphoma and myeloproliferative neoplasm (MPN) (**Table 1**). In the following content, we focus on elaborating on the biological functions of CAFs in the above-mentioned diseases based on current research progress, hoping to provide reference for the search for potential therapeutic targets.

**Table 1:** The role of CAFs in hematological malignancies and possible molecular mechanisms.

Disease types	Source of CAFs	Activation factors	Makers	Biological function	Potential mechanisms	Reference
AML	BM-MSCs	TGF-β1	FSP-1,α-SMA, FAP, collagen I/III	Reduce the therapeutic sensitivity of cytarabine (Ara-C)	growth differentiation factor 15	Zhai et al. (134)
	BM-MSCs	–	FAPα	Reduce the therapeutic sensitivity of Ara-C	β-catenin signaling pathway	Mei et al. (135)
ALL	BM-MSCs and MSC cell line HS27a	exposed to Ara-C and daunorubicin	α-SMA, CAF-like chemokine and cytokine	Reduce the therapeutic sensitivity of Ara-C	mitochondrial transfer	Burt et al. (136)
	human choroid plexus fibroblasts	co-culture with leukemia cells	α‐SMA, vimentin, PDGFRB, PDPN, VEGFA, pro-inflammatory cytokines and chemokines	–	–	Fernández-Sevilla et al. (137)
	BM-MSCs	TGF-β	α-SMA, FAP	promote leukemia cells migration and invasion	–	Pan et al. (138)
	BM-MSCs	TGF-β	α-SMA, FAP	promote leukemia cells growth and invasion	SDF-1/CXCR4, FN/integrin α5β1	Pan et al. (139)
	stromal cells	–	CAF/EGR_high_, CAF/EGR_low_	CD4 T-cell proliferation	FGF7-FGF1 and PDGFA-PDGFRA/B signaling	Joo et al. (140)
CLL	BM-MSCs	miR-146a	α-SMA, FAP	–	–	Yang et al. (141)
	BM-MSCs and endothelial cells	CLL-derived exosomes	CXCL1, IL6, IL34, CCL2, ICAM1, and MMP1	create a niche that promotes the adhesion, survival, and growth of CLL cells	–	Paggetti et al. (142)
	BMSC lines HS-5 and NKtert, and two immortalized CAFs cell lines from two patient-derived pancreatic cancer specimens	–	PDGFRA/B, FAP, ITGB1, PDPN, CAV1, IL6, IL1A, LIF, CSF3, CXCL1, ACTA2, CTGF, COL1A1	facilitate leukemic survival	LYN kinase regulates the polarization of fibroblasts towards inflammatory cancer-associated phenotype	Vom et al. (143)
MM	osteoclasts and MSCs	myeloma cells	FAP	support myeloma cells survival	–	Ge et al. (144)
	BM-MSCs	tumor cells conditioned medium or RPMI8226 cells	FAP	Reduce the therapeutic sensitivity of bortezomib	β-catenin signaling pathway	Zi et al. (145)
	ECs, HSPCs, MSCs	active MM CAFs and MM cells	FSP-1, α-SMA, FAP	induce MM cell proliferation and prevent apoptosis	SDF1α and integrins	Frassanito et al. (146)
	BM mononuclear cells	–	refer to reference (146)	Reduce the therapeutic sensitivity of bortezomib	activate autophagy	Frassanito et al. (147)
	BM stromal cells	–	α-SMA, FSP-1, CD45^−^	promote MM cells proliferation and invasion	u-PA/u-PAR system	Ciavarella et al. (148)
	bone marrow mononuclear cells	–	α-SMA, FAP	promote MM angiogenesis	CAF-derived exosome microRNA-21	Miaomiao et al. (80)
Lymphoma	fibroblasts from patient biopsy samples	–	α-SMA	support the survival of primary lymphoma cells and induce drug resistance	enhancing glycolysis, exosomes	Kunou et al. (149)
	fibroblasts from patient sample	–	α-SMA^+^ and CD31^-^	support tumor cells survival	release pyruvate	Sakamoto et al. (150)
	fibroblasts from primary lymph nodes	–	CD29, CD73, CD90, and CD105 positive, CD45, CD146, and CD271 negative	Reduce the therapeutic sensitivity of Brentuximab-Vedotin	direct fibroblasts contact	Bankov et al. (151)
	fibroblasts from MF tissues and normal skin	–	FAPα	Reduce the therapeutic sensitivity of doxorubicin and increases tumor cells migration	CXCL12/CXCR4	Aronovich et al. (152)
	primary human lymphatic fibroblasts	–	α-SMA, FAP α	immunosuppression	up-regulate PD-L1	Apollonio et al. (153)
MPN	MSCs	LOXL2	α-SMA, FAP	associate with the grading of reticulin fibrosis	–	Liu et al. (154)
	healthy mesenchymal stem cells HS5	IGFBP-6, purmorphamine	α-SMA, FAP, TGF-β	alter the microenvironment of PMF	IGFBP-6/SHH/TLR4 axis	Longhitano et al. (155)

-, not reported.

### 7.1 CAFs promote the progression of acute leukemia

Acute leukemia (AL) is a common malignant tumor type in hematological system. Most patients with AL achieve complete remission after induction chemotherapy. However, the prognosis of quite a few high-risk patients is still poor, and the long-term survival is not optimistic. Therefore, the new treatment strategy provides great hope for further improving the efficacy of leukemia. During the past few years, the significant rise of immunotherapy has revolutionized the treatment of leukemia.

Recently, studies have shown that the TME has played a key role in leukemia progression (10–12, 156–159). The TME is a contributing factor to the failure or success of leukemia treatment, which may lead to a shift in treatment methods and concepts. CAFs are important stromal cells in TME, and their role in AL has been gradually revealed, which is expected to become a potential therapeutic target for leukemia.

Research on AML has shown that the interaction between leukemia cells and bone marrow niche can affect hematopoietic function in AML, promote leukemia cell survival, and lead to chemotherapy resistance (157, 160–162). For CAFs in AML microenvironment, Zhai et al. (134) found that CAFs co-cultured with AML cell lines could significantly prolong the survival of leukemia cells, reduce apoptosis, and lower the sensitivity of leukemia cells to chemotherapy drugs. Growth differentiation factor 15 (GDF15) secreted by CAFs may be an important factor for the protective effect of CAFs-mediated chemotherapy. Targeting or down-regulating GDF15 can significantly improve the sensitivity of leukemia cells to chemotherapy drugs. Another study demonstrated that the high expression of the CAFs marker FAPα in bone marrow stromal cells reduced the sensitivity of leukemia cells to cytarabine by stimulating the β-catenin signaling pathway (135). Suggesting that CAFs and its marker FAPα in AML microenvironment may be an important factor for the poor prognosis of AML patients.

ALL is one of the common types of leukemia. Research has shown that there are abnormally activated stromal cells in the bone marrow of patients with ALL, which can form specific stromal niches to protect leukemia cells from chemotherapy drug damage (163). Targeting TME is an important direction in the treatment of ALL (156). The study performed by Burt et al. (136) on ALL found that MSCs could be activated into CAFs under the stimulation of daunorubicin or cytarabine to promote the progression of leukemia. Moreover, co-culturing leukemia cells with human choroid plexus fibroblasts could enable the latter to obtain CAFs phenotype (137). Meanwhile, our previous research found that MSCs in the ALL microenvironment acquired CAFs phenotype, which played an essential role in promoting ALL cell migration and invasion (138). The SDF-1/C-X-C chemokine receptor type 4 (CXCR4) axis, as a signaling axis facilitating the interaction between tumor cells and stromal cells, promotes the integration α5β1 on leukemia cells to bind to FN in MSCs with CAFs phenotype, thus stimulating the interaction between ALL cells and MSCs with CAFs phenotype, which may be a potential molecular mechanism that fosters the progression of ALL. Application of CXCR4 inhibitor AMD3100 or targeting down-regulation of integrin β1 can weaken the promoting effect of MSCs with CAFs phenotype on leukemia cell migration and invasion (139).

Adult T-cell leukemia/lymphoma (ATLL) refers to a rare aggressive T-cell malignant tumor caused by human T-cell leukemia virus type 1 infection. Joo et al. (140) employed single-cell RNA sequencing and T-cell receptor clone analysis to dissect different cell types, and identified a new subset of CAFs showing abundant EGFR-related transcripts, including early growth response 1 and 2 (EGR1 and EGR2). Further study showed that CAFs in ATLL exerted an essential role in CD4 T cell proliferation through FGF7-FGF1 and PDGFA-PDGFRA/B signaling.

Based on the above research, CAFs promote leukemia progression to varying degrees. To this end, Li et al. (164) constructed a leukemia-associated fibroblastic tumor cell line HXWMF-1. This provides a convenient way to further explore the role of CAFs in leukemia.

### 7.2 CAFs promote the progression of chronic lymphoblastic leukemia

Chronic leukemia is a type of malignant tumor which is originated from bone marrow hematopoietic stem cells, including CLL and chronic myeloid leukemia. Studies on CAFs in chronic leukemia mainly concentrate on CLL, which is a clonal malignant disease of lymphocytes, and the survival and proliferation of tumor cells cannot be separated from the surrounding stromal microenvironment. In the TME of CLL, the activation of CAFs is mainly dependent on CLL-derived factors (165). Co-culturing CLL cells with MSCs can promote the acquisition of CAFs phenotype in MSCs (166). Yang et al. (141) showed that exosomes secreted by leukemia cells could transport miR-146a to MSCs, and miR-146a transported to MSCs promoted the transformation of MSCs into CAFs by targeting the down-regulation of ubiquitin-specific peptidase 16. In Paggetti’s study (142), CLL-derived exosomes facilitated the acquisition of CAFs phenotype by fusing with BM-MSCs and endothelial cells, and further accelerated the disease progression of CLL. LYN protein is a non-receptor tyrosine protein kinase, belonging to the Src family of kinases. A recent study showed that it was overexpressed in fibroblasts of lymph nodes in CLL patients, which could regulate the polarization of fibroblasts towards inflammatory cancer-associated phenotype, therefore promoting leukemia survival (143). These results suggest that stromal cells in the CLL microenvironment can be activated to CAFs in response to the stimulation of leukemia cells, which may be a key component in the study of the pathogenesis of CLL.

### 7.3 CAFs promote the progression of multiple myeloma

CAFs exerts a vital role in the biobehavior of MM, and myeloma cells can induce MSCs differentiate into CAFs in a dose-dependent manner (167). Ge et al. (144) found that the FAP, a marker of CAFs, was highly expressed in MM bone marrow and could promote the growth of myeloma cells. Zi et al. (145) also showed similar results, and demonstrated that FAP could protect MM cells from apoptosis induced by bortezomib via β-catenin signaling pathway. In another study, Frassanito et al (146) showed that the expression levels of CAFs markers (FSP-1, α-SMA, FAP) in bone marrow of patients with active MM were obviously higher than those in patients with MM remission, patients with monoclonal gammopathy of undetermined significance, and patients suffering from iron deficiency anemia. In the MM microenvironment, activated CAFs promote the chemotaxis, adhesion, proliferation and reduce apoptosis of MM cells through cytokine signaling and intercellular contact. In further studies, the research team found that CAFs were insensitive to bortezomib treatment and protected MM cells from bortezomib-induced apoptosis. Bortezomib can trigger CAFs to secrete high levels of IL-6, IL-8, insulin-like growth factor (IGF)-1 and TGF-β, induce reactive oxygen species and activate autophagy in bortezomib resistant CAFs to reduce treatment sensitivity (147). Ciavarella et al. (148) also suggested that CAFs promoted the proliferation and invasion potential of MM cells, and inhibition of urokinase plasminogen activator receptor (u-PAR) gene expression in CAFs significantly weakened the above biological effects. CAFs-derived exosomes are also a key component promoting the progression of MM, and CAF-derived exosome miR-21 can enter MM endothelial cells in a time-dependent manner and initiate angiogenesis by promoting proliferation, migration, and tubule formation (80).

Considering role of CAFs in the progression of MM, the use of recombinant human erythropoietin as a potential therapeutic strategy can inhibit cell proliferation of MM patient-derived CAFs while increasing CAFs apoptosis (168). Recently, study has also shown that CAFs can hinder the anti-tumor activity of CAR-T cells and promote MM progression, further demonstrating that dual target CAR-T cell therapy targeting both MM cells and CAFs can significantly improve the therapeutic efficacy of CAR-T cell therapy (169). These results indicate that CAFs exert a vital role in the MM microenvironment, and targeting CAFs may be an effective intervention to improve the therapeutic efficacy of MM patients.

### 7.4 CAFs promote the progression of lymphoma

Lymphoma is one of the most common malignancies in blood system, including non-Hodgkin lymphoma (NHL) and Hodgkin lymphoma (HL). In lymphoma TME, CAFs can support lymphoma cell survival by increasing glycolysis and induce drug resistance in lymphoma cells by secreting exosomes (149). CAFs also promote the survival of lymphoma cells by secreting pyruvate (150). Hodgkin and Reed Sternberg (HRS) cells are characteristic cells of HL. Studies have demonstrated that HRS cells adhered to fibroblasts are usually protected from damage induced by the therapeutic drug Brentuximab Vedotin (151). FAP is one of the common markers of CAFs. Jin et al. (170) found through ^68^Ga-FAPI PET/CT that the uptake of FAP in HL lesions increased and was correlated with the intensity of immunostaining. In aggressive NHL lesions, FAP has moderate to severe immunostaining, while in indolent NHL lesions, FAP staining is weaker. Fungal granuloma (MF) is a primary cutaneous T-cell lymphoma (CTCL). Mehdi et al.’s (171) study also showed that the expression of CAFs marker FAP α increased with disease staging. Co-culture of MF fibroblasts with CTCL cell line MyLa cells could increase the expression of IL16 and IL4 in MyLa cells, and inhibit the expression of Th1 markers IFNG and TBX21. Additionally compared with MF fibroblasts, normal fibroblasts inhibited MKI67 expression in MyLa cells. Another study revealed that CAFs protected MF cells from doxorubicin-induced cell death and increased their migration by secreting CXCL12 (152). It is suggested that monitoring FAP expression may be a way to characterize lymphoma. Diffuse large B-cell lymphoma is the most common type of NHL. Apollonio et al. (153) found that co-culturing primary human lymphoid fibroblasts (HLF) with DLBCL cell lines induced the expression of CAFs markers FAP α and α-SMA in HLF, and exhibited significant changes in the cytoskeleton and matrix remodeling ability, while up-regulating PD-L1 expression and driving immune suppression. In further research, the research team found that DLBCL tumor cells also converted fibroblast reticular cells (FRCs) into immunosuppressive CAFs. Lymphoma FRCs can exhibit CAFs like immunophenotypes, including FAP, α-SMA, and upregulation of immune regulatory MHC class I, PD-L1, and PD-L2 molecules (172). For therapeutic interventions, the study performed by Aoki et al. (173) showed that the compound emetine hindered the potential of CAFs to support tumor cell viability *in vitro* and significantly inhibit tumor growth *in vivo*. Additionally, emetine induced cell death in primary refractory lymphoma cells with MYC rearrangement. Collectively, these findings suggest that CAFs may be a key factor in the progression of lymphoma.

### 7.5 CAFs in myeloproliferative neoplasm

MPN is a clonal and chronic disease characterized by myeloid cell proliferation. Regarding the study of CAFs in MPN, Schmitt-Graeff et al. (174) showed that the expression of CAFs marker α-SMA was up-regulated in MPN, and its expression increased with the aggravation of fibrosis degree. In previous studies, our research group also found that CAFs markers were expressed to varying degrees in MPN, among which, the expression of α-SMA, FAP, and lysyl-oxidase-like 2 (LOXL2) was related to the degree of bone marrow fibrosis. *In vitro* cell experiments demonstrated that the use of recombinant human LOXL2 significantly increased the expression of α-SMA and FAP in MSCs, suggesting that LOXL2 might be capable of stimulating MSCs to obtain the CAFs phenotype (154). Insulin like growth factor binding proteins (IGFBPs) are a family of six highly homologous protein members with high affinity for IGF. Longhitano et al. (155) found that IGFBP-6 expression significantly increased in primary myelofibrosis (PMF), and IGFBP-6 stimulated the up-regulation of CAFs markers α-SMA, FAP, and TGF-β expression in human bone marrow stromal cells HS5, suggesting that IGFBP-6 could stimulate the transformation of MSCs into CAFs and might be correlated with the progression of myelofibrosis. However, there is currently no detailed report on the mechanism by which CAFs exert a role in MPN.

**Figure 3:**
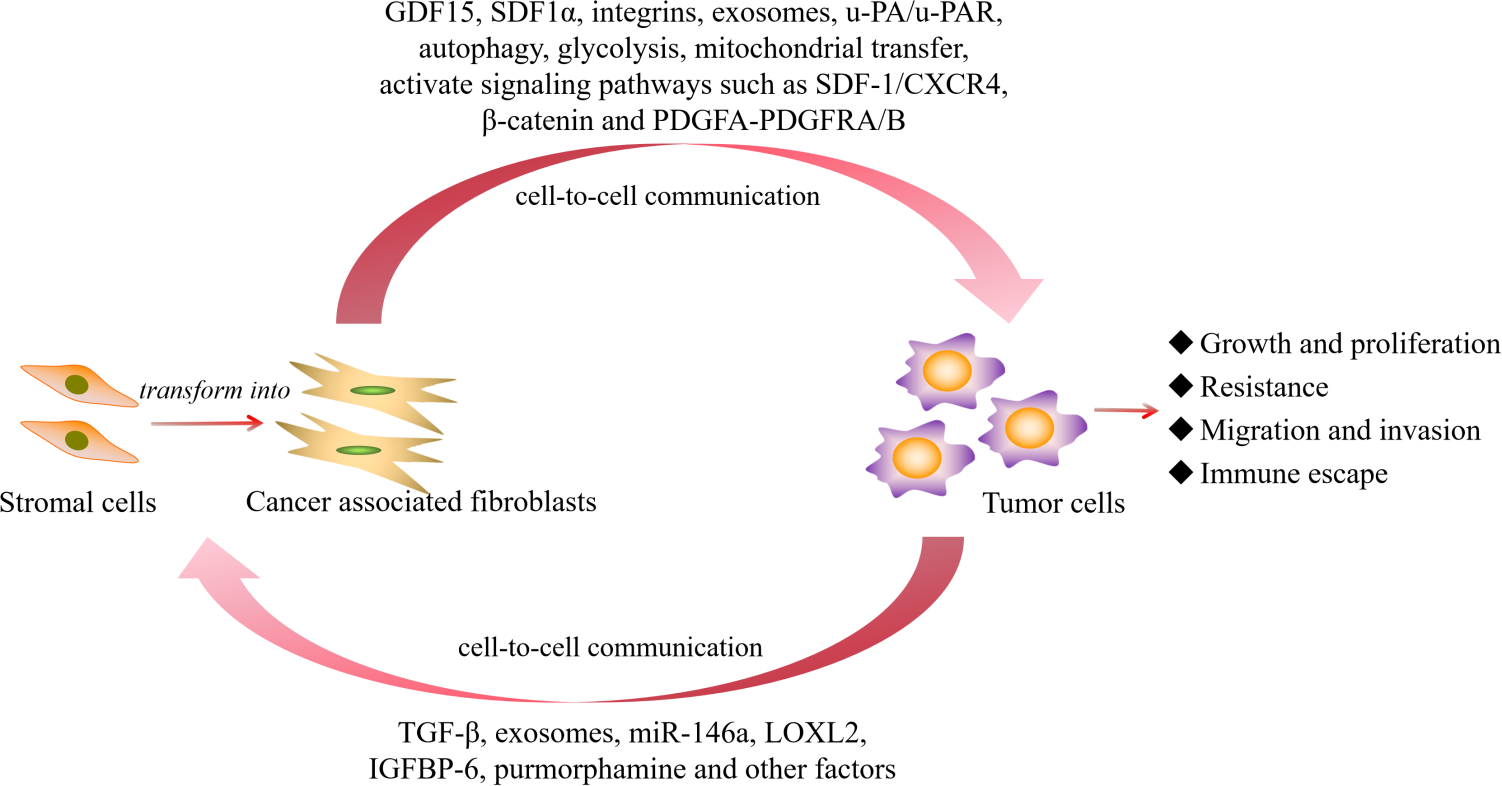
The activation of CAFs and the main molecular pathways that promote the progression of hematological malignancies. GDF15, growth differentiation factor 15; SDF1α, stromal cell-derived factor-1 α; u-PA/u-PAR, urokinase plasminogen activator/urokinase plasminogen activator receptor; PDGF, platelet-derived growth factor; TGF-β, transforming growth factor β; LOXL2, lysyl-oxidase-like 2; IGFBP-6, insulin like growth factor binding protein 6.

**Figure 4:**
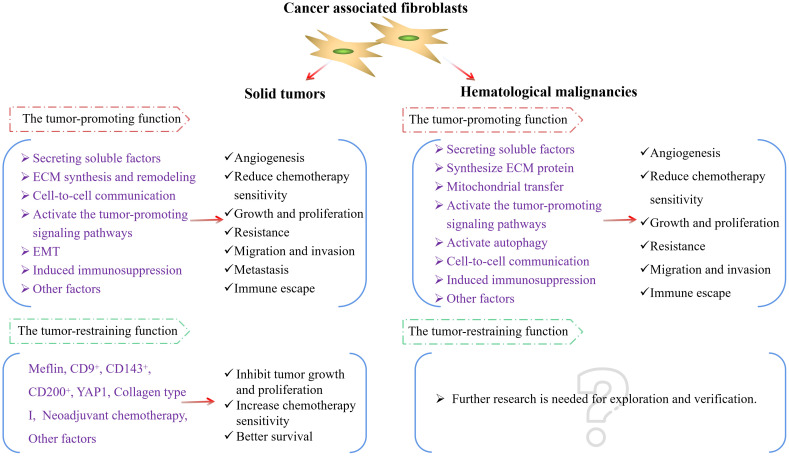
CAFs in solid tumors and hematological malignancies. ECM, extracellular matrix; EMT, epithelial-mesenchymal transition; YAP1, Yes-associated protein 1.

In the aforementioned study, we found that CAFs mainly play a tumor-promoting role in hematological malignancies. Whether they possess certain tumor-suppressing subtypes or tumor-inhibitory biological functions remains to be further explored and demonstrated. In light of this, we have summarized the activation of CAFs and the main molecular pathways through which they promote the progression of hematological malignancies (**Figure 3**), and compared the different roles played by CAFs in solid tumors and hematological malignancies (**Figure 4**) to make the research situation of CAFs in tumors more visually clear.

## 8 Strategies for targeting CAFs

For the therapeutic strategies targeting CAFs, many studies have conducted corresponding explorations. Through exploring the various related published research works, a series of studies and explorations are currently focused on inhibiting the poor prognostic markers of CAFs, inactivating CAFs, targeting the signaling pathway activated by CAFs and its downstream factors, or preventing the interaction between tumor cells and CAFs (42, 59, 175).

FAP is an important marker of CAFs. The study performed by Akai et al. (176) showed that near-infrared light immunotherapy (NIR-PIT) targeting FAP effectively induced CAFs-specific cell death without damaging adjacent normal cells. At the same time, the use of FAP inhibitor Talabostat (PT100) might also be one of the options to improve the efficacy of tumor treatment (177). The CXCR4/SDF-1 axis exerts an important role in coordinating tumor cells and CAFs (32). The use of CXCR4 inhibitor AMD3100 may be one of the strategies to reduce the interaction between CAFs and tumor cells and then improve therapeutic efficacy (139). Bao et al. ‘s (178) study revealed that the combination of FAP-targeted radiopharmaceutics [^177^Lu] Lu-DOTAGA.(SA.FAPi)_2_ and AMD3100 significantly inhibited cell proliferation, migration and colony formation in triple-negative breast cancer cells, and showed synergistic effects on the 4T1 tumor models, while reducing the number of bone marrow-derived suppressor cells. It is suggested that [^177^Lu] Lu-DOTAGA.(SA.FAPi)_2_ combined with AMD3100 may be an effective treatment for tumor. Ripretinib is a potent receptor tyrosine kinase inhibitor. Mori et al. (179) showed that in the presence of the chemotherapy drug carboplatin, ripretinib could prevent CAFs survival and inhibit the proliferation of ovarian clear cell carcinoma. The use of the flavonoids Oroxylin A (OA) can also inactivate CAFs and hinder the proliferation and invasion of tumor cells (180). Meanwhile, appropriate digoxin can inhibit the sub-population of tumor stem cells and the production of CAFs cytokines in the CAFs-tumor cell co-culture system, and digoxin combined with chemotherapy can improve the therapeutic effect of tumor (181). Low dose digoxin can inhibit the expression of TGF-β-induced CAFs marker fibronectin expression without producing adverse cytotoxicity (30). In addition, 1,25 (OH) 2D3 can inhibit the activation of CAFs in tumors (182). It is indicated that the combination of conventional chemotherapy and CAFs targeting drugs may be a potential strategy to enhance the therapeutic effect of tumor treatment. In addition, The use of dual targeting strategy to simultaneously target tumor cells and CAFs may be an effective treatment option (169, 183).

Moreover, multiple clinical trials have been conducted to explore the potential impact of intervention and targeting CAFs on the therapeutic efficacy of tumor treatment. Among them, the clinical trials of the therapy targeting CAFs marker FAP (NCT05442151, NCT05641896, NCT04621435, etc.) are currently underway. Some clinical experiments have been completed, but no research results have been posted (NCT05547321, NCT04857138, NCT05043714). As CXCR4/SDF-1 is a key signaling axis mediating the interaction between CAFs and tumor cells, several clinical studies targeting CXCR4 have also been carried out. A phase 2 clinical trial using CXCR4 antagonist BL-8040 and pembrolizumab in metastatic pancreatic patients (NCT02907099) showed that the application of BL-8040 and pembrolizumab could increase the quantity of T cell infiltration in tumor tissues, but was accompanied by 26.67% severe adverse events and 33.33% non-severe adverse events. In hematological malignancies, several clinical trials using CXCR4 antagonists or targeting CXCR4 have also been conducted, but some completed clinical trials (NCT01120457, NCT04274738, NCT01236144, NCT01010880) have not posted experimental results. A recent phase 1 clinical trial of CXCR4 modified B-cell maturation antigen CAR-T for relapsed/refractory MM is currently recruiting, but no preliminary results have been presented (NCT04727008). Moreover, several clinical trials using targeting downstream signaling pathways of CAFs (NCT01333475, NCT02392572) and intervention of their adverse prognostic secretory factors (TGF-β, PDGFR, VEGF, MMP, etc.) have also been conducted (NCT02423343, NCT02146222, NCT02202746, NCT00033215, NCT00001683), expecting to directly or indirectly consume CAFs, reduce or eliminate their tumor-promoting characteristics to improve the therapeutic efficacy of tumor treatment. However, the results of some clinical trials are not satisfactory and are accompanied by varying degrees of adverse events. At present, there are still some clinical trials underway, with the expectation that the therapeutic efficacy based on the intervention of CAFs will be further verified in more types of tumors.

Overall, although a number of basic and clinical trial studies on the treatment of CAFs have been carried out, due to the various subtypes and mechanisms of CAFs in different tumors, there is currently no unified treatment target. Additionally, although multiple clinical trials have been conducted, some results are not satisfactory and are accompanied by varying degrees of adverse events, which may be a challenge for current treatment. This requires further exploration and argumentation in future research.

## 9 The heterogeneity of CAFs needs attention

In the aforementioned content, we mentioned that CAFs can originate from various stromal cells in the TME, and can promote tumor progression through multiple mechanisms and signaling pathways. However, they also possess the biological function of inhibiting tumor progression. Simultaneously, CAFs may present different subtypes in different tumor or different stages of the tumor (43, 175, 184–186). These results reveal the heterogeneity of CAFs in the TME.

To explore the composition of bone marrow stromal cells, Baryawno et al. (187) performed single-cell RNA sequencing on non-hematopoietic bone marrow cells of C57Bl/6 mice and found 17 subtypes of stromal subsets, including fibroblast subpopulations consisting of 5 clusters. Fibroblasts-1 and 2 expressed progenitor cell markers CD34 and MSCs markers, but did not express endothelial and pericyte genes. Fibroblasts-3, 4, and 5 correlated with tendon/ligament cells. They jointly express Sox9 and TF Scleraxis. This indicates the existence of different subtypes of fibroblasts in the bone marrow microenvironment. In addition, numerous studies on single-cell sequencing analysis have revealed that CAFs can exhibit distinct subtypes, markers, and biological functions in tumors and different organs (44, 188–191). In recent years, an increasing number of CAFs subtypes have been discovered and defined, which makes CAFs more heterogeneous.

Meanwhile, CAF markers may exert differential biological functions in tumors, and undergo subtype changes during the tumor progression process. α-SMA is an important marker of CAFs, and studies have suggested that its high expression can promote tumor progression (33, 52). However, there were also study showing that depletion of α-SMA^+^ myofibroblasts leads to enhanced EMT and cancer stem cells, and low myofibroblasts in tumors is associated with poor survival (192). In the study of cervical cancer, recent research performed by Bueno-Urquiza et al. (46) showed that CAFs exhibited a myofibroblast like phenotype (CAF α-SMA^+^FAP^+^) in the early stages of cervical cancer, while in the late stages, they exhibited a primitive phenotype (CAF α-SMA^-^ FAP^+^). In the study of pancreatic ductal adenocarcinoma, Tao et al. (193) identified NFs and nine different subtypes of CAFs, and found that the CAFs subtypes exhibited plasticity in differentiation, transitioning from early normal-like CAFs (nCAFs) to iCAFs and myCAFs, ultimately leading to more invasive proliferative CAFs (pCAFs). In addition, in breast cancer, Kashyap et al. (194) found that when CAFs conditioned medium from different subtypes of breast cancer and different breast cancer cell lines were cultured and treated with chemotherapy drugs, there exited differences in the abundance of CAFs secreted proteins in each group, suggesting that there were heterogeneous CAFs populations in the microenvironment of different cancer subtypes. In the study of intrahepatic cholangiocarcinoma, Hu et al. (195) showed that CAFs could be categorized into cancer suppressive or cancer promoting types (rCAFs or pCAFs). Among them, polycomb group ring finger 4 (PCGF4) promoted cell migration, drug resistance activity and stem cell characteristics. rCAFs triggered proteasome-dependent degradation of PCGF4, while pCAFs enhanced the stability of PCGF4 by activating the IL-6/p-STAT3 pathway.

Apart from the fact that different CAFs subtypes may lead to the heterogeneity of CAFs, the role that CAFs play in promoting or inhibiting tumor growth in different tumors is also one of the characteristics that reflect their heterogeneity. Just as we mentioned in the previous content. These findings suggest that CAFs from different tumor types or different CAFs sources may have variable typical markers and present different phenotypes and functions. Researchers need to focus on these issues in the study process to more accurately explore the possible role of CAFs in different tumors and contribute to finding potential treatment strategies.

## 10 Discussion and conclusion

TME is a complex and dynamic microenvironment that supports the survival and proliferation of tumor cells. The occurrence, development and metastasis of tumors are closely related to the internal and external environments in which tumor cells are located. This includes not only the structure, function and metabolism of the tissue where the tumor is located, but also the intrinsic environment surrounding the tumor cells. The interaction between the two enables TME to adapt to support the survival, growth and metastasis of tumors.

CAFs, as an important stromal cell component in the TME, play a significant role in the occurrence and development of tumors. In this review, we summarize and elaborate on the origin, markers, characteristics, and research progress and treatment strategies of CAFs in solid tumors and hematological malignancies, and emphasize the heterogeneity of CAFs. Based on literature reports, we found that the activation of CAFs in the TME can originate from multiple stromal cells and has various markers and biological subtypes. In the research of solid tumors, most studies have found that they play an important role in promoting tumor angiogenesis, growth, proliferation, and mediating tumor resistance, invasion, and immune escape. However, some studies have also reported that CAFs can have biological subtypes and functional characteristics that inhibit tumor progression. Recently, based on single-cell sequencing studies, more biological subtypes of CAFs have been revealed, and these different subtypes of CAFs further reveal the heterogeneity and diversity of the functions of CAFs. In the research of hematological malignancies, the possible roles of CAFs have been revealed in diseases such as AML, ALL, CLL, MM, lymphoma and MPN. Most of them have exerted tumor-promoting biological functions. Whether CAFs have more biological subtypes and whether they have the function of inhibiting tumor progression require further exploration and verification in the future.

Regarding the therapeutic strategies targeting CAFs, studies have achieved satisfactory results in basic research. However, in clinical trials, the intervention treatments targeting CAFs still face many issues that need to be addressed, including long-term efficacy, drug safety, and adverse events.

In conclusion, CAFs are not only considered to be a vital factor for promoting tumor progression and leading to tumor immune escape in solid tumors, but also a key component in promoting tumor progression in hematological malignancies. It interacts with tumor cells, secretes cytokines, promotes tumor EMT, promotes ECM remodeling, and directly interacts with tumor cells, thereby promoting tumor cell growth and proliferation, and mediating the process of tumor drug resistance and invasion. Blocking the interaction between CAFs and tumor cells may enhance the sensitivity of tumor cells to chemotherapeutic drugs, and reduce the proliferation and invasion of tumor cells.

Based on this, targeting CAFs might be an important therapeutic measure to improve TME and reduce tumor progression. However, during the research process, we need to pay attention to the heterogeneity of CAFs, as well as the inhibitory subtypes and functions of CAFs on tumors. Precisely targeting the tumor-promoting subtypes of CAFs or inhibiting CAFs activation and inhibiting the interaction between CAFs and tumor cells might be one of the effective options for improving tumor treatment strategies in the future. Overall, an increasing number of studies are still needed to demonstrate the therapeutic efficacy of targeted CAFs to accurately achieve the purpose of improving the long-term survival of tumors.
